# Differences in the prevalence of physical activity and cardiovascular risk factors between people living at low (<1,001 m) compared to moderate (1,001–2,000 m) altitude

**DOI:** 10.3934/publichealth.2021050

**Published:** 2021-08-31

**Authors:** Martin Burtscher, Grégoire P Millet, Jeannette Klimont, Johannes Burtscher

**Affiliations:** 1 University of Innsbruck, A-6020, Innsbruck, Austria; 2 Institute of Sport Sciences, University of Lausanne, CH-1015, Lausanne, Switzerland; 3 Department of Biomedical Sciences, University of Lausanne, CH-1015, Lausanne, Switzerland; 4 Unit Demography and Health, Directorate Social Statistics, Statistics Austria, 1110 Vienna, Austria

**Keywords:** altitude, health, residence, lifestyle, physical activity, age, sex

## Abstract

Living at moderate altitude (up to about 2,000 m) was shown to be associated with distinct health benefits, including lower mortality from cardiovascular diseases and certain cancers. However, it remains unclear, whether those benefits are mainly due to environmental conditions (e.g., hypoxia, temperature, solar ultra-violet radiation) or differences in lifestyle behavior, including regular physical activity levels. This study aims to compare altitude-related differences in levels of physical activity and the prevalence of cardiovascular risk factors such as obesity, hypertension, hypercholesterolemia, and diabetes in an Alpine country. We interrogated the Austrian Health Interview Survey (ATHIS) 2019, a nationally representative study of persons aged over 15 years living in private Austrian households. The results confirm a higher prevalence of hypertension (24.2% vs. 16.8%) in men living at low (<1,001 m) compared to those at moderate (1,001 to 2,000 m) altitude. Women living above 1,000 m tend to have a lower prevalence of hypercholesterolemia (14.8% vs. 18.8%) and diabetes (3.2% vs. 5.6%) than their lower living peers. Both sexes have lower average body mass index (BMI) when residing at moderate altitude (men: 25.7, women: 23.9) compared to those living lower (26.6 and 25.2). Severe obesity (BMI > 40) is almost exclusively restricted to low altitude dwellers. Only men report to be more physically active on average when living higher (1,453 vs. 1,113 weekly MET minutes). These novel findings confirm some distinct benefits of moderate altitude residence on heath. Beside climate conditions, differences in lifestyle behavior, i.e., physical activity, have to be considered when interpreting those health-related divergences, and consequently also mortality data, between people residing at low and moderate altitudes.

## 1. Introduction

Living at the moderate altitudes (up to about 2,000 m) in alpine areas of Switzerland [1] and Austria [2] have been reported to be associated with certain health benefits. These specifically include a reduced mortality from cardiovascular and cerebrovascular diseases but also from some cancers. In the Swiss population, mortality from coronary heart disease was shown to decrease by 22% per 1000 m and from stroke by 12% per 1,000 m gain in altitude (up to about 2000 m) [1]. We demonstrated that age-standardized mortality rates (ASMR) in Austria decreased almost linearly from low (<251 m) to higher (1,001 to about 2,000 m) altitudes by 45% for male colorectal cancer and 38% for female breast cancer [2]. Recently, analyzing all deaths across 10 years in Austria, we also reported that residents at moderate altitude (1,001–2,000 m) had lower ASMRs from circulatory diseases and cancer, when compared to low (<251 m) residents, also exhibiting some sex-specific differences [3].

In those studies, altitude-specific climate conditions, i.e., hypoxia, temperature and solar radiation, have been assumed to constitute the primary factors for the reported mortality benefits. Altitude-related data on physical activity, the probably most important protective lifestyle factor [4],[5] amongst others (e.g., nutrition), body mass index (BMI) and associated cardiovascular risk factors are largely lacking. Differences in physical activity, however, could importantly contribute to the benefits attributed to altitude. We hypothesized that residents in moderate altitude—at least in the alpine areas—have a higher level of physical activity, than their low-altitude counterparts, that contributes to the prevalence reduction of cardiovascular risk factors such as obesity, hypertension, hypercholesterolemia, and diabetes. If this were the case, it would indicate that greater importance should be attached to the consideration of lifestyle behaviors as contributors to health benefits reported for moderate-altitude dwellers. Thus, the aim of the present study was to evaluate altitude-dependent and sex-specific differences of physical activity behavior, BMI, and associated cardiovascular risk factors between people living at low (<1,001 m) or moderate (1,001–2,000 m) altitude in Austria. As data from such moderate altitudes are largely lacking, a representative population-based health-interview survey was interrogated for that purpose.

## 2. Methods

We compared altitude-related differences in levels of physical activity and the prevalence of obesity and other cardiovascular risk factors (hypertension, hypercholesterolemia, and diabetes) using the recordings of the Austrian Health Interview Survey (ATHIS) 2019 [7]. ATHIS, is a nationally representative study of persons aged 15 years and more living in private Austrian households.

The survey included a total of 15,461 people (extrapolated 7.4 Mio persons) of which 3.6% were living above 1,000 m. Variables of interest (age in 5-year groups; prevalence of hypertension, hypercholesterolemia, diabetes; BMI, and habitual physical activity (weekly metabolic equivalent, MET minutes) are presented descriptively for both sexes living at low (<1,001 m) or moderate altitude (1,001 to about 2,000 m).

All data were self-reported. The presence of a risk factor was answered affirmatively, if the diagnosis was made by a physician. BMI was calculated from the reported body mass and stature. Different domains of physical activity such as “work-related physical activity”, “transportation (commuting) physical activity” and “sports, fitness recreational (leisure) physical activity” and the duration were recorded. The answers should refer to a typical week. Here, we report the amount of physical activity in MET minutes or MET hours (=60 MET minutes). Resting energy expenditure is defined as 1 MET (1 metabolic equivalent corresponding to 3.5 mL oxygen consumption per minute per kg body mass). For example, walking or jogging at a pace requiring 5 METs per hour (60 minutes) corresponds to 300 MET minutes. As public health guidelines recommend a minimum of weekly 150–300 minutes moderate intensity aerobic physical activity or 75–150 minutes of vigorous intensity physical activity [8], at least 900 MET minutes are necessary to reach those minimum levels (moderate intensity activity of at least 3 METs for 300 minutes).

The indicated statistical differences (unpaired t-tests, Mann-Whitney-U-Test or chi-squared tests) refer to comparisons between values recorded at the low and moderate altitude level. Logistic-regression analysis was used to estimate adjusted odds ratios and their 95% confidence intervals of independent predictive variables for the occurrence of hypertension, hypercholesterolemia, or diabetes. All available independent variables have been entered into the analyses in a single step (enter method). P-values below 0.05 were considered statistically significant.

## 3. Results

Average values (and standard deviations) of BMI and physical activity (Met minutes/week), as well as numbers of the prevalence of obesity, hypertension, hypercholesterolemia, and diabetes, are shown for the total sample population and 2 age groups (<51 years and >50 years) living below an altitude of 1,001 m and above 1,000 m (Table 1), and separately for both sexes (Table 2).

Within the total sample, the amount of regular physical activity is higher, BMI values and the prevalence of hypertension are lower in those living above 1,000 m compared to those below 1,001 m. The age group below 51 years is primarily contributing to those differences (Table 1).

The prevalence of hypertension is significantly higher in men living below 1001 m compared to those above 1,000 m. Women, living above 1,000 m tend (p < 0.1) to have a lower prevalence of hypercholesterolemia and diabetes than their lower living peers (Table 2).

**Figure 1.**
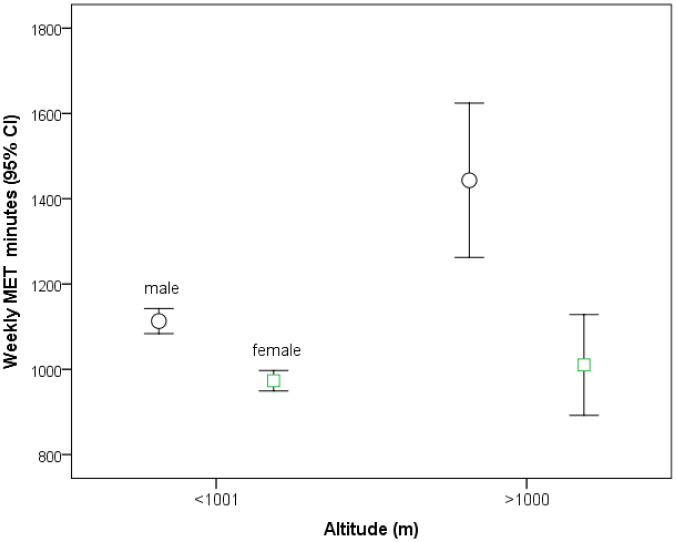
Regular physical activity (weekly MET minutes and 95% confidence intervals) for men and women living at low and moderate altitude.

Women have lower average BMI values than males at both altitude levels, but the average BMI of both sexes is lower at altitudes higher than 1,000 m. On average men are more physically active than women regardless of the altitude of residence. Only men, but not women, living at higher altitudes report a larger amount of physical activity compared to their lower living peers (Table 2, Figure 1).

**Figure 2.**
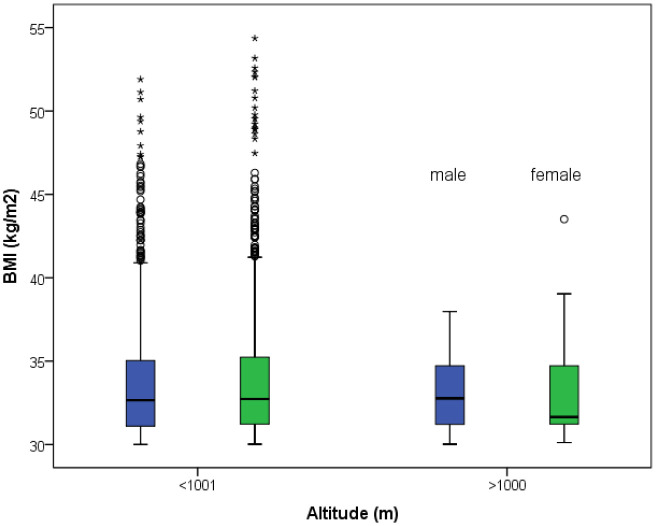
Distribution of subjects with body mass index (BMI) > 30 kg/m^2^ (n = 2,534) at an altitude below 1,001 m and above 1,000 m for men and women. Medians are not different between altitudes for both sexes, but class 3 (severe) obesity is almost exclusively present at low altitude.

When only taking obese people (BMI > 30 kg/m^2^) into account, medians are not different between altitudes for both sexes, but class 3 (severe) obesity is almost exclusively restricted to people living below 1,001 m (Figure 2). Moreover, all considered risk factors are significantly more prevalent in obese people compared to those with a BMI equal or below 30; hypertension 44 vs. 19%, hypercholesterolemia 28% vs. 17%, diabetes 14% vs. 5%; (all p-values < 0.001).

**Table 1.** Characteristics of the study population (total and 2 age groups).

Altitude	<1,001 m			>1,000 m			P-value <1,001 m vs. >1,000 m		
Study population	Total	<51 years	>50 years	Total	<51 years	>50 years	Total	<51 years	>50 years
Number	14903	6926	7977	558	268	290			
Age (5-year groups)	7.7 (3.9)	4.2 (1.2)	10.7 (12.2)	7.8 (4.0)	4.3 (2.1)	10.9 (2.2)	0.61		
Hypertension (%)	23.5	5.7	39.0	19.4	2.6	34.8	0.02	0.03	0.16
Hypercholesterolemia (%)	19.1	7.7	29.1	17.0	6.7	26.6	0.23	0.64	0.36
Diabetes (%)	6.2	1.1	10.7	5.9	1.1	10.3	0.85	0.76	0.92
BMI (kg/m^2^)	25.8 (4.8)	24.8 (4.8)	26.7 (4.6)	24.8 (4.1)	23.9 (3.9)	25.6 (4.1)	<0.001	0.004	0.046
PA (MET minutes/week)	1038 (1169)	928 (1029)	1133 (1271)	1229 (1311)	1193 (1310)	1262 (1312)	<0.001	<0.001	0.17

Note: BMI: body mass index; PA: physical activity; MET: metabolic equivalent—presented as mean values ± standard deviation.

**Table 2.** Characteristics of the study population by sex. Nationally representative study of persons aged over 15 years living in private Austrian households.

Altitude	<1,001 m			>1,000 m			P-value <1,001 m vs. >1,000 m	
Study population	males	females	P-value	males	females	P-value	males	females
Number	6892	8011		274	284			
Age (5-year groups)	7.6 (3.8)	7.8 (3.9)	0.008	7.9 (3.9)	7.6 (4.0)	0.44	0.31	0.53
Hypertension (%)	24.2	23.0	0.07	16.8	21.8	0.13	0.005	0.66
Hypercholesterolemia (%)	19.5	18.8	0.24	19.3	14.8	0.15	0.93	0.09
Diabetes (%)	7.0	5.6	0.001	8.8	3.2	0.005	0.25	0.07
BMI (kg/m^2^)	26.6 (4.9)	25.2 (5.0)	<0.001	25.7	23.9	<0.001	0.001	<0.001
PA (MET minutes/week)	1113 (1246)	973 (1096)	<0.001	1453	1013	<0.001	<0.001	0.58

Note: BMI: body mass index; PA: physical activity; MET: metabolic equivalent—presented as mean values ± standard deviation.

**Figure 3.**
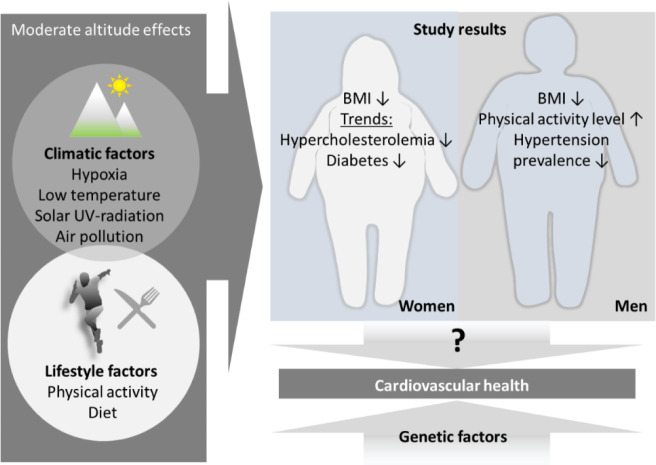
Schematic presentation of the main findings from the Austrian Health Interview Survey of living at moderate altitude and factors potentially promoting observed effects.

**Table 3.** Predictors for the occurrence of hypertension, hypercholesterolemia, or diabetes derived from logistic regression analyses.

Risk factor	Predictors	Odds ratio (95% CI)	P-value
Hypertension	Age (per 5 years)	1.36 (1.34–1.38)	<0.001
	Sex (female)	1.03 (0.94–1.13)	0.51
	BMI (per unit)	1.14 (1.13–1.15)	<0.001
	Altitude (per 1,000 m)	0.75 (0.62–0.89)	0.002
	Physical activity (per MET hour/week)	0.99 (0.99–1.01)	0.36
	Hypercholesterolemia (yes)	2.10 (1.90–2.31)	<0.001
	Diabetes (yes)	1.95 (1.67–2.28)	<0.001
Hypercholesterolemia	Age (per 5 years)	1.16 (1.15–1.18)	<0.001
	Sex (female)	0.97 (0.89–1.06)	0.49
	BMI (per unit)	1.04 (1.03–1.05)	<0.001
	Altitude (per 1,000 m)	0.82 (0.69–0.97)	0.02
	Physical activity (per MET hours/week)	1.00 (0.99–1.01)	0.70
	Hypertension (yes)	1.97 (1.78–2.17)	<0.001
	Diabetes (yes)	1.84 (1.60–2.13)	<0.001
Diabetes	Age (per 5 years)	1.26 (1.23–1.29)	<0.001
	Sex (female)	0.75 (0.65–0.86)	<0.001
	BMI (per unit)	1.10 (1.09–1.12)	<0.001
	Altitude (per 1,000 m)	0.64 (0.4–0.86)	0.003
	Physical activity (per MET hours/week)	0.99 (0.98–0.99)	<0.001
	Hypertension (yes)	2.07 (1.77–2.41)	<0.001
	Hypercholesterolemia (yes)	1.09 (1.81–2.42)	<0.001

Note: BMI: body mass index; MET: metabolic equivalent; CI: confidence interval.

Multiple logistic regression analyses revealed that age, BMI, altitude, and the 2 other risk factors (as independent variables) are predictors for the risk factors “hypertension”, “hypercholesterolemia”, and “diabetes”. Whereas 1 unit (kg/m^2^) gain in BMI is associated with an increased risk of hypertension/hypercholesterolemia/diabetes by 14%/4%/10%, 1,000 m gain in altitude is associated with a risk reduction by 25%/18%/36% (Table 3). In addition, the weekly amount of physical activity and female sex were associated with a lower diabetes prevalence.

## 4. Discussion and conclusions

The findings from the Austrian Health Interview Survey confirm the existence of specific, age- and sex-dependent differences regarding the amount of regular physical activity and the prevalence of obesity and cardiovascular risk factors between people living at lower (<1,001 m) and higher altitudes (1,001 up to about 2,000 m), but regular physical activity does not importantly predict cardiovascular risk factors (hypertension, hypercholesterolemia, diabetes) (Figure 3). These results are of particular relevance for the interpretation of potential mechanisms responsible for health benefits, including the lower mortality rates, observed at the moderate altitudes of alpine regions.

### 4.1. Potential relevance of altitude-dependent differences and effects of physical activity

In this study, men and women on average met minimal requirements of physical activity at low and moderate altitudes. Men are significantly more physically active than women, and men living at moderate altitude are even more active than men living at low altitudes. These differences may contribute to the reported beneficial effects of moderate altitude on reduced all-cause mortality [3], and that from cardiovascular and cerebrovascular diseases [1] as well as from certain cancers [2]. The higher level of physical activity in men (at moderate altitude vs. low altitude and compared to women) might be associated with the more favorable effects on the altitude-related cardiovascular mortality in males (−21%) compared to females (−11%) [3]. The present analyses did not reveal essential effects of the amount physical activity (MET minutes) on the prevalence of selected cardiovascular risk factors, i.e., hypertension, hypercholesterolemia, diabetes, which may be partly due to the fact that minimum recommendations for physical activity have on average been met by those living at low and moderate altitude as well. However, physical activity may elicit its beneficial effects on longevity by the higher level of exercise capacity, which was shown to be a more powerful predictor of mortality in men than other established risk factors for cardiovascular disease [9]. Nevertheless, men at moderate altitude may benefit from their higher physical activity levels, for which a favorable health impact is well established. For instance, pooled data from six studies in Europe and the United States (including 661,137 adults), convincingly demonstrated that meeting minimum recommendations for physical activity was associated with a large longevity benefit [10]. These authors reported further benefits on longevity until reaching a threshold at a level 3 to 5 times above that of minimum recommendations. Moreover, men benefited more from vigorous and women more from moderate intensity physical activity, and the mortality reduction from cardiovascular diseases was more pronounced when compared to that from cancer [10]. A recent study (including 527,662 adult participants; 32% women) confirmed favorable effects of sufficient regular physical activity on all major cardiovascular risk factors, more pronounced for diabetes and obesity compared to hypertension and hypercholesterolemia, also exhibiting some slight sex differences [11].

The present study displays significantly lower BMI in both sexes living at moderate compared to low altitude, and severe obesity (BMI > 40) seems to be a phenomenon occurring almost only in low altitude populations in this study. These results are in line with previous observation of lower obesity rate in moderate altitude residents [12]. On this matter, the respective influences of the environmental vs life-style or behavioral factors are not disentangled [13]. Particularly, the altitude-induced changes in energy balance due to both energy expenditure (i.e., higher resting metabolic rate and physical activity energy expenditure, as shown in the present study) and energy intake (i.e., lower appetite) remain unclear [14].

Not surprisingly, all accompanied cardiovascular risk factors were significantly more prevalent in obese people (BMI > 30). The close association between obesity and cardiovascular disease events, including mortality, has recently been affirmed by meta-analyses, highlighting once again risk-modulating capacity of the individual cardiorespiratory fitness [15]. Growing evidence from preclinical and clinical data indicates that obesity may also worsen the incidence, severity, and mortality from several types of cancers, e.g., breast cancer [16]. Low amounts of physical activity represent a strong and independent predictor for obesity, favoring the development of a self-perpetuating vicious circle (increasing obesity reduces capacity to perform exercise, which in turn promotes obesity, etc.), finally resulting in severe obesity [19]. Based on our findings this vicious circle may be more easily disrupted when living at moderate altitude. Further multidisciplinary research is required to investigate the main reasons of this observation and how healthy public policies may contribute to improve the physical activity level of people who live in these mountainous areas. In summary, altitude-related and sex-specific differences in the amount of regular physical activity and the individual BMI, especially severe obesity, must be considered when interpreting effects of living at moderate altitude on health factors.

### 4.2. Potential relevance of altitude-dependent differences and effects of climate conditions

Beside lower BMI values, moderate altitude turned out to be an independent predictor for lower prevalence of cardiovascular risk factors, i.e., hypertension, hypercholesterolemia and diabetes (Table 3). Moderate altitude affects health by complex interactions between lifestyle factors (e.g., physical activity as discussed above; nutrition, etc.), genetic factors [20] socioeconomic and in particular environmental (climatic, pollution, etc.) conditions.

Environmental factors (e.g., hypobaric hypoxia, increased solar radiation, low ambient temperature, reduced air pollution), have hitherto been assumed (although mostly derived from higher altitudes) to be the key factors for the protective roles of altitude in several diseases [3],[21]. Numerous studies indicate lower incidence- and mortality-rates from cardiovascular diseases when living at higher altitude [1],[22],[23], reduced cancer mortality [2],[24],[25], but also decreased risk for the development of diabetes mellitus [26], obesity [12]–[14],[27] and metabolic syndrome [28],[29].

With increasing elevation, the barometric pressure and the related partial pressures of inspiratory, alveolar and arterial oxygen decrease. The decline in the arterial oxygen saturation according to the oxygen-hemoglobin dissociation curve is modest at the moderate altitudes considered in the present study (1,001–2,000 m). However, oxygen desaturation may become more pronounced during conditions like sleep or physical activity [30],[31]. Consequently, hypoxia-related activation of molecular responses, revolving notably around the hypoxia-inducible factor (HIF) pathways, can be initiated even at those moderate altitudes, resulting in the expression of dozens of genes in order to maintain tissue oxygen supply [32]. This might affect the development and progression of various diseases, including cardiovascular diseases and cancer [1],[21],[24],[25],[33]. It has also been suggested that HIF activation leads to alterations in appetite by increasing leptin gene expression [34], basal metabolic rate, and reductions in body adiposity [14],[35], thereby potentially contributing to the demonstrated lower obesity prevalence in this study. One may speculate that the two most important health factors attributed to altitude residence are in fact mediated by the increased physical activity level and the lower obesity prevalence.

However, some environmental factors could actually play a role. On the one hand, cold exposure (even at moderate altitude) during work, exercise, etc. is unavoidable, likely contributing to blood pressure elevation in certain individuals [36], especially at higher altitudes [37]. On the other hand, it may result in cold adaptations/habituations. Such adaptations encompass, e.g., blunted sympathetic stress responses and improved stress tolerance but may also elevate the basal metabolic rates and thereby counteract obesity and associated development of diabetes [26],[38],[39].

It has further been suggested that the higher levels of solar ultra-violet (UV) radiation at moderate altitude might benefit the human organism, e.g., by favoring cardiovascular health and reducing cancer development, likely at least partially mediated by the role of UV in vitamin D synthesis [40],[41].

Taken together, beside lifestyle factors, various climate conditions may independently and in concert contribute to sex-specific health effects of living at moderate altitude.

### 4.3. Limitations

Although the total sample size of the underlying dataset is large, the number of people living at moderate altitude (1,001–2,000 m) is relatively small. This is due to the fact that the present dataset is based on a population-based representative study and comprises the rather small moderate-altitude (as compared to low-altitude) population in Austria. Moreover, given that the analyzed data are self-reported, a reporting bias cannot be excluded. Nevertheless, to the best of our knowledge this is the first nationally representative study evaluating potential differences in regular physical activity and the prevalence of obesity and other major cardiovascular risk factors between people living at low and moderate altitude of the Alps.

In conclusion, the presented findings from the Austrian Health Interview Survey (2019) confirm differences in altitude-dependent regular physical activity levels and the prevalence of cardiovascular risk factors. Specifically, men living at moderate altitude (1,001–2,000 m) are more physically active compared to those living at lower altitudes. At moderate altitude, the BMI of both sexes is lower and severe obesity is essentially non-existent. While the prevalence of hypertension is lower in men, hypercholesterolemia and diabetes tend to be lower in female altitude dwellers. These findings are of importance for the interpretation of altitude-related health effects, including mortality data.
